# Glances at the Hospitals

**Published:** 1905-02-25

**Authors:** 


### GLANCES AT THE HOSPITALS.

### BEDFORD COUNTY HOSPITAL.

THE income for the year amounted to £4,921, and there was a balance of £98 left over from the previous year, making a sum of £5,019. The expenditure was £5,168. Nearly £4,000 was received in donations, and Mr. Barnard presented the hospital with a Finsen light apparatus. Colonel and Mrs. Dillon gave a steriliser for the operating theatre. There were 79 patients in residence on the first of January, and 876 were admitted during the year, making a total of 955 under treatment. Of these, 514 were discharged recovered; 275 were discharged relieved; 28 not relieved; 58 died; and 80 remained in the wards. Of the 58 deaths, 9 took place within 24 hours of admission. The average cost of each in-patient was £4 19s. 11d.; the average cost per bed occupied was £65 7s. 8d.; and the average stay of each patient in the hospital was 28 days. The Roentgen Ray Department was used in 148 cases, 120 of these being fractures, dislocations, or other injuries. The medical and surgical tables are properly drawn out, and in all cases show the numbers "cured," "relieved," "not relieved," and "died." The operation table is equally accurate. There were 14 operations for appendicitis, death following in 2 suppurative cases. Twenty-eight amputations were performed, all being successful. The total number of operations was 640, and of these 468 were perfectly successful; 136 partly so; 6 not; and death followed in 14 cases. Chloroform was given in 589 instances; A.C.E. mixture in 21; ether in 20; and cocaine in 10.

### WORCESTER GENERAL INFIRMARY.

THE income for the year, excluding legacies, but including donations, was £4,654, and the expenditure was £7,222, showing a deficiency of £2,568. To meet this deficit the committee have been under the necessity of selling stock which had been looked on as a permanent investment, and they say that this process will have to be repeated unless the income considerably increases. To us it seems as if the workpeople's own subscriptions ought to be susceptible of considerable increase as at present they appear to be under £200. The daily average number of patients in residence was 96, and the cost per bed was £52 4s. There were in the hospital being trained 34 probationers. Of these 7 completed their training, 2 were unsuitable, one was promoted to be sister, and 24 remained. On the first of January there were 93 patients in residence, and 985 were admitted up to the 31st of December, making a total of 1,078 under treatment. Of these 534 were cured, 296 were relieved 52 not relieved, 86 died, and 110 remained in the wards. The medical and surgical tables are properly compiled showing the results of treatment, but the operation table merely states the names of the operations and the numbers operated on. The total number was 521. Ether was the anæsthetic in 371 cases, chloroform in 137, ethyl chloride in 28, A.C.E. mixture in 32, and gas in 4. We are glad to be able to record that there is a bacteriological report which shows that 162 examinations were made: 14 for diphtheria, 37 for tubercle, 5 for anthrax, 19 for typhoid, and 87 for other organisms.

